# Impact of rewarming rate on the mortality of patients with accidental hypothermia: analysis of data from the J-Point registry

**DOI:** 10.1186/s13049-019-0684-5

**Published:** 2019-11-26

**Authors:** Makoto Watanabe, Tasuku Matsuyama, Sachiko Morita, Naoki Ehara, Nobuyoshi Miyamae, Yohei Okada, Takaaki Jo, Yasuyuki Sumida, Nobunaga Okada, Masahiro Nozawa, Ayumu Tsuruoka, Yoshihiro Fujimoto, Yoshiki Okumura, Tetsuhisa Kitamura, Bon Ohta

**Affiliations:** 1 Department of Emergency Medicine, Kyoto Prefectural University of Medicine, Kamigyo-ku, Kyoto, 602-8566 Japan; 2 Senri Critical Care Medical Center, Saiseikai Senri Hospital, Suita, Japan; 3 Department of Emergency, Japanese Red Cross Kyoto Daiichi Hospital, Kyoto, Japan; 4 Department of Emergency Medicine, Rakuwa-kai Otowa Hospital, Kyoto, Japan; 5 Department of Emergency and Critical Care Medicine, Japanese Red Cross Society Kyoto Daini Red Cross Hospital, Kyoto, Japan; 6 Department of Emergency Medicine, Uji-Tokushukai Medical Center, Uji, Japan; 7 Department of Emergency Medicine, North Medical Center, Kyoto Prefectural University of Medicine, Kyoto, Japan; 8 Department of Emergency and Critical Care Medicine, National Hospital Organization, Kyoto Medical Centre, Kyoto, Japan; 9 Department of Emergency and Critical Care Medicine, Saiseikai Shiga Hospital, Ritto, Japan; 10 Department of Emergency and Critical Care Medicine, Kidney and Cardiovascular Center, Kyoto Min-iren Chuo Hospital, Kyoto, Japan; 11 Emergency and Critical Care Medical Center, Osaka City General Hospital, Osaka, Japan; 12 Department of Emergency Medicine, Fukuchiyama City Hospital, Fukuchiyama, Japan; 13 Division of Environmental Medicine and Population Sciences, Department of Social and Environmental Medicine, Graduate School of Medicine, Osaka University, Osaka, Japan

**Keywords:** Accidental hypothermia, Rewarming, Rewarming rate

## Abstract

**Background:**

Accidental hypothermia (AH) is defined as an involuntary decrease in core body temperature to < 35 °C. The management of AH has been progressing over the last few decades, and numerous techniques for rewarming have been validated. However, little is known about the association between rewarming rate (RR) and mortality in patients with AH.

**Method:**

This was a multicentre chart review study of patients with AH visiting the emergency department of 12 institutions in Japan from April 2011 to March 2016 (Japanese accidental hypothermia network registry, J-Point registry). We retrospectively registered patients using the International Classification of Diseases, Tenth Revision code T68: ‘hypothermia’. We excluded patients whose body temperatures were unknown or ≥ 35 °C, who could not be rewarmed, whose rewarmed temperature or rewarming time was unknown, those aged < 18 years, or who or whose family members had refused to join the registry. RR was calculated based on the body temperature on arrival at the hospital, time of arrival at the hospital, the documented temperature during rewarming, and time of the temperature documentation. RR was classified into the following five groups: ≥2.0 °C/h, 1.5–< 2.0 °C/h, 1.0–< 1.5 °C/h, 0.5–< 1.0 °C/h, and < 0.5 °C/h. The primary outcome of this study was in-hospital mortality. The association between RR and in-hospital mortality was evaluated using multivariate logistic regression analysis.

**Result:**

During the study, 572 patients were registered in the J-Point registry, and 481 patients were included in the analysis. The median body temperature on arrival to the hospital was 30.7 °C (interquartile range [IQR], 28.2 °C–32.4 °C), and the median RR was 0.85 °C/h (IQR, 0.53 °C/h–1.31 °C/h). The in-hospital mortality rates were 19.3% (11/57), 11.1% (4/36), 14.4% (15/104), 20.1% (35/175), and 34.9% (38/109) in the ≥2.0 °C/h, 1.5–< 2.0 °C/h, 1.0–< 1.5 °C/h, 0.5–< 1.0 °C/h, and < 0.5 °C/h groups, respectively. Multivariate regression analysis revealed that in-hospital mortality rate increased with each 0.5 °C/h decrease in RR (adjusted odds ratio, 1.49; 95% confidence interval, 1.15–1.94; *P*_trend_ < 0.01).

**Conclusion:**

This study showed that slower RR is independently associated with in-hospital mortality.

## Background

Accidental hypothermia (AH) is defined as an involuntary decrease in core body temperature to < 35 °C [1]. AH cases are frequently observed in the emergency department and can present significant problems. A previous study has stated that the mortality rate of patients with AH was as high as approximately 30% [2]. Severe hypothermia (body core temperature < 28 °C) was specifically associated with a high risk of sudden cardiac arrest [3]. The management of AH has been progressing over the last few decades, and numerous techniques for rewarming have been validated [4–6]; however, little is known about the optimal rewarming rate (RR).

Theoretically, it seems reasonable to rewarm patients with AH as fast as possible to avoid the fatal complications of hypothermia such as cardiac instability [1, 5]. Conversely, rewarming is associated with a number of complications: for example, hypotension, neutropenia, thrombocytopenia, electrolyte changes, cardiac arrhythmias, gastrointestinal bleeding, and infection [4, 6–9]. These complications may consequently affect the mortality rate as a number of deaths in patients with AH have been noted after successful rewarming [6]. Hence, the selection of appropriate rewarming strategies, including RR, is considered the major problem in AH. Suggestions about RR vary among studies; some studies suggest the benefit of rapid RR [4, 5, 10], while other studies do not [6, 11–13]. These suggestions are based on small observational studies, animal studies, and studies in cardiac surgery. Thus, existing guidelines do not mention the optimal RR because of insufficient evidence [1, 3].

We performed the Japanese accidental hypothermia network registry (J-Point registry), a multicentre retrospective observational study, which enrolled 481 adult patients with AH. Using this registry, we evaluated the association between RR and in-hospital mortality.

## Methods

### Study design and setting

We conducted a multicentre chart review study of patients with AH visiting the emergency departments of 12 institutions in Japan (Japanese accidental hypothermia network registry [J-Point registry]). The Japanese AH network comprises eight critical care medical centres (CCMCs) and four non-CCMCs with an emergency department across the Kyoto, Osaka, and Shiga Prefectures in Japan. For the participating institutions, the median annual emergency department visit volume was 19,651 (interquartile range [IQR], 13,281–27,554). Using these data, we evaluated the association between RR and mortality.

### Participants

We retrospectively registered patients using the International Classification of Diseases, Tenth Revision (ICD-10) code T68: ‘hypothermia’ from April 2011 to March 2016. We excluded patients whose body temperatures were unknown or ≥ 35 °C, who could not be rewarmed, whose rewarmed temperature or rewarming time was unknown, aged < 18 years, or those who or whose family members had refused to join the registry.

### Data collection and quality control

The details of the methodology were described previously [14]. In summary, all chart reviewers were emergency physicians who underwent training for appropriate data extraction. A predefined uniform datasheet was used for data collection.

Collected baseline patient characteristics were as follows: sex, age, activities of daily living (ADLs) before the emergency department visit (independent, needing some assistance, or needing total assistance), residence (living at home alone, living at home but not alone, nursing home, or homeless), medical history (cardiovascular disease, neurological disease, endocrine disease, psychiatric disease, malignant disease, or dementia, or others), location (indoor or outdoor), and mode of arrival (walk-in or transported using an ambulance).

The data collected upon arrival at the hospital were as follows: vital signs upon arrival at the hospital (body temperature, blood pressure, heart rate, and Glasgow Coma Scale [GCS] score), biological data (serum pH, bicarbonate [HCO_3_-] [mEq/L], lactate [mmol/L], sodium [mEq/L], potassium [mEq/L], and glucose [mg/dL] levels), cold exposure, associated conditions, treatment process, and outcome. The presence of cold exposure, which is a possible cause of the hypothermia, was determined by the clinician who cared for the patient or who entered the data of this study. The Sequential Organ Failure Assessment (SOFA) score was only calculated for patients admitted to the intensive care unit. Consistent with a previous study [2], associated conditions were classified into internal disease, traumatic injury, alcohol intoxication, drowning, and self-harm, and others. The diagnoses of internal disease were obtained from ICD-9 or ICD-10 code in the medical records. Rewarming procedures were divided into active external/minimally invasive rewarming (warm intravenous fluids, warm blanket, forced warm air, heating pads, and warm bath) and active internal rewarming (lavage, intravascular haemodialysis, and extracorporeal membrane oxygenation) [15]. Other treatment information included endotracheal intubation, use of catecholamines, and emergent transvenous cardiac pacing. The data collected on the outcomes were in-hospital mortality and the incidence of ventricular fibrillation or pulseless ventricular tachycardia (VF/VT).

### Outcome measures

In this study, the primary outcome was in-hospital mortality, and the secondary outcome was the incidence of VF/VT. We calculated RR based on the body temperature upon arrival, documented temperature during rewarming, and time spent for the rewarming. We evaluated the association between RR and these outcomes.

### Statistical analysis

Patients were divided into the following five groups according to their RR: ≥2.0 °C/h, 1.5–< 2.0 °C/h, 1.0–< 1.5 °C/h, 0.5–< 1.0 °C/h, and < 0.5 °C/h. Patients’ characteristics, in-hospital information, and outcomes were evaluated between the five groups using Kruskal-Wallis tests for continuous variables and Fisher’s exact tests for categorical variables. For the post-hoc analyses of these tests, Steel-Dwass multiple comparison tests and Bonferroni correction for multiple comparisons were used, respectively. Regarding the primary outcome, the association between each category of the RR and in-hospital mortality was evaluated using the univariate and multivariate logistic regression analyses, with crude or adjusted odds ratios (AORs) and their 95% confidence interval (CI) as the effect variables. In multivariate models, we selected the potential confounders that were considered to be associated with the clinical outcomes, including sex, age category (adults aged 18–64 years, the young–elderly aged 65–74 years, or the elderly–elderly aged ≥75 years), body temperature at arrival to the hospital (mild [≥32 °C], moderate [28–32 °C], and severe [< 28 °C]), the number of past medical history (none, one, or multiple), ADLs (independent, need for some assistance, need for total assistance, or unknown), systolic blood pressure (cardiac arrest, unmeasurable, 40–90 mmHg, or 90 < mmHg), cold exposure (yes, no, or unknown), presence of associated internal diseases (yes or no), and active internal rewarming (yes or no). Regarding the secondary outcome, the association between each category of the RR and occurrence of VF/VT was evaluated using the univariate logistic regression analyses, with crude odds ratios and their 95% CI as the effect variables. Additionally, we divided the patients into subgroups according to age category (adults aged 18–64 years, elderly patients aged ≥65 years), location (indoor, outdoor), body temperature at arrival to the hospital (mild, moderate, severe), associated conditions (presence of associated internal disease or not), and rewarming procedure (use of active internal rewarming or not). The association between each category of the RR and in-hospital mortality was evaluated in the same way as the primary outcome. All *P* values were two-sided, and 0.05 levels were considered statistically significant. All statistical analyses were performed using the Statistical Package for the Social Sciences software (V.24 J), R (The R Foundation for Statistical Computing, version 3.30, Saitama, Japan), and EZR (Saitama Medical Center, Jichi Medical University, version 1.32, Saitama, Japan), which is a graphical user interface for R [16].

## Result

**Fig. 1:**
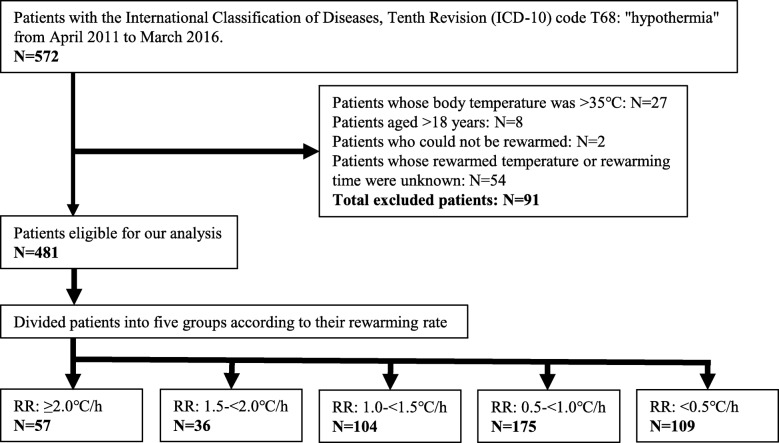
Study flowchart

During the study period, 572 patients were registered in the J-Point registry; out of which, 27 patients whose body temperature was ≥35 °C, 8 patients aged < 18 years, 2 patients who could not be rewarmed, and 54 patients whose rewarmed temperature or rewarming time were unknown were excluded in the study (Fig. 1). We finally enrolled 481 patients for the analysis.

### Patient characteristic and in-hospital data

The baseline patients’ characteristics are presented in Table 1. Approximately half of the patients were male (50.5%), and the median age was 79 years (IQR, 67–87 years). Overall, 78% of the patients had AH in an indoor setting. Patients were younger in the ≥2.0 °C/h group. Patients in the < 0.5 °C/h groups were more likely to have decreased ADLs and lived in nursing homes. There was no significant difference in the number and types of past medical history among the five groups.

**Table 1:** Baseline characteristics of the study population according to their rewarming rate category

	All patients	Rewarming rate (°C/h)					*P* values*
		(≥2.0)	(1.5–< 2.0)	(1.0–< 1.5)	(0.5–< 1.0)	(< 0.5)	
	*n* = 481	*n* = 57	*n* = 36	*n* = 104	*n* = 175	*n* = 109	
Men	243 (50.5)	25 (43.9)	14 (38.9)	56 (53.8)	93 (53.1)	55 (50.5)	0.424
Age, y, median (IQR)	79 (67–87)	71 (64–81)^a, b^	77 (68–85)	76 (64–87)	82 (70–87)^a^	81 (72–89)^b^	0.002
Age category							
Adults aged 18–64 years	101 (21.0)	15 (26.3)	8 (22.2)	27 (26.0)	35 (20.0)	16 (14.7)	0.240
Young-elderly aged 65–74 years	79 (16.4)	18 (31.6)^a^	7 (19.4)	18 (17.3)	20 (11.4)^a^	16 (14.7)	0.014
Elderly-elderly aged ≥75 years	301 (62.6)	24 (42.1)^a, b^	21 (58.3)	59 (56.7)	120 (68.6)^a^	77 (70.6)^b^	0.002
Activity of daily living							
Independent	335 (70.0)	49 (86.0)^a^	30 (83.3)^b^	74 (71.2)	121 (68.4)	61 (56.0)^a, b^	< 0.001
Need for some assistance	114 (23.7)	8 (14.0)	5 (13.9)	28 (26.9)	44 (25.3)	29 (26.6)	0.183
Need for total assistance	31 (6.4)	0 (0.0)^a^	1 (2.8)	2 (1.9)^b^	10 (6.3)^c^	18 (16.5)^a, b, c^	< 0.001
Unknown	1 (0.21)	0 (0.0)	0 (0.0)	0 (0.0)	0 (0.0)	1 (0.9)	
Residence							
Home	432 (89.8)	53 (93.0)	34 (94.4)	101 (97.1)^a^	156 (89.1)	88 (80.7)^a^	0.002
Living alone	193 (40.1)	17 (29.8)	19 (52.8)	51 (49.0)^a^	75 (42.9)	31 (28.4)^a^	0.004
Living not alone	239 (49.7)	36 (63.2)	15 (41.7)	50 (48.1)	81 (46.3)	57 (52.3)	0.180
Nursing home	30 (6.2)	0 (0.0)^a^	1 (2.8)	1 (1.0)^b^	11 (6.3)	17 (15.6)^a, b^	< 0.001
Homelessness	4 (0.8)	0 (0.0)	1 (2.8)	0 (0.0)	2 (1.1)	1 (0.9)	0.496
Unknown	15 (3.1)	4 (7.0)	0 (0.0)	2 (1.9)	6 (3.4)	3 (2.8)	
Location							
Indoor	375 (78.0)	36 (63.2)^a, b^	27 (75.0)	68 (65.4)^c, d^	150 (85.7)^a, c^	94 (86.2)^b, d^	< 0.001
Outdoor	106 (22.0)	21 (36.8)^a, b^	9 (25.0)	36 (34.6)^c, d^	25 (14.3)^a, c^	15 (13.8)^b, d^	< 0.001
Mode of arrival							
Ambulance	453 (94.2)	55 (96.5)	35 (97.2)	101 (97.1)	166 (94.9)	96 (88.1)	0.065
Walk-in	28 (5.8)	2 (3.5)	1 (2.8)	3 (2.9)	9 (5.1)	13 (11.9)	0.065
Past medical history							
Cardiovascular disease	213 (44.3)	27 (47.4)	13 (36.1)	37 (35.6)	77 (44.0)	59 (54.1)	0.070
Neurological disease	86 (17.9)	7 (12.3)	4 (11.1)	21 (20.2)	31 (17.7)	23 (21.1)	0.507
Endocrine disease	116 (24.2)	16 (28.1)	8 (22.2)	18 (17.3)	44 (25.1)	30 (27.5)	0.391
Psychiatric disease	110 (22.9)	18 (31.6)	11 (30.6)	24 (23.1)	37 (21.1)	20 (18.3)	0.261
Malignant disease	50 (10.4)	7 (12.3)	3 (8.3)	9 (8.7)	23 (13.1)	8 (7.3)	0.546
Dementia	99 (20.6)	5 (8.8)	7 (19.4)	20 (19.2)	39 (22.3)	28 (25.7)	0.111
Other	88 (18.7)	7 (12.3)	6 (16.7)	19 (18.3)	35 (20.0)	21 (19.3)	0.784
Unknown	7 (1.46)	1 (1.8)	0 (0.0)	1 (1.0)	3 (1.7)	2 (1.8)	1.000
Number of past medical history							
None	118 (24.5)	13 (22.8)	11 (30.6)	28 (26.9)	47 (26.9)	19 (17.4)	0.308
One	152 (31.6)	18 (31.6)	11 (30.6)	37 (35.6)	50 (28.6)	36 (33.0)	0.803
Multiple	211 (43.9)	26 (45.6)	14 (38.9)	39 (37.5)	78 (44.6)	54 (49.5)	0.464

Values are expressed numbers (percentages) unless indicated otherwise

Groups that share a superscript letter were significantly different from post-hoc pairwise comparisons

* Represents P for heterogeneity across the 5 rewarming rate groups. Comparisons between the 5 groups were evaluated with Kruskal-Wallis test for numeric variables and Fisher’s exact test for categorical variables. For post-hoc pairwise comparisons of these tests, Steel-Dwass multiple comparison tests and Bonferroni correction for multiple comparisons were used, respectively

The in-hospital data are presented in Table 2. The median body temperature was 30.7 °C (IQR, 28.2–32.4 °C), and the median RR was 0.85 °C/h (IQR, 0.53–1.31 °C/h). Most of the patients received active external or minimally invasive rewarming, whereas about one in six patients received active internal rewarming. The percentage of patients with cardiac arrest was highest in the > 2.0 °C/h group. Acid-base status abnormality (pH, lactate, and HCO_3_-) are more common in the > 2.0 °C/h group, but serum sodium and potassium levels did not differ among the five groups. There was no significant difference in GCS and SOFA score. The prevalence of internal disease association was significantly high in the < 0.5 °C/h group.

**Table 2:** In-hospital data of the study population according to their rewarming rate category

	All patients	Missing	Rewarming rate (°C/h)					*P* values*
			(≥2.0)	(1.5–< 2.0)	(1.0–< 1.5)	(0.5–< 1.0)	(< 0.5)	
	*n* = 481		*n* = 57	*n* = 36	*n* = 104	*n* = 175	*n* = 109	
Body temperature, Median (IQR)	30.7 (28.2–32.4)	0 (0.0)	27.2 (25.6–31.2)^a, b^	28.8 (26.0–30.6)^c,^	29.8 (27.5–32.0)^a^	30.9 (28.8–32.5)^b, c^	32.2 (30.2–33.6)^a, b, c^	< 0.001
Body temperature category		0 (0.0)						
Mild (≥32 °C)	163 (33.9)		10 (17.5)^a^	5 (13.9)^b^	27 (26.0)^c^	60 (34.3)^d^	61 (56.0)^a, b, c, d^	< 0.001
Moderate (28–32 °C)	216 (44.9)		15 (26.3)^a^	16 (44.4)	48 (46.2)	92 (52.6)^a^	45 (41.3)	0.011
Severe (< 28 °C)	102 (21.2)		32 (56.1)^a^	15 (41.7)^b^	29 (27.9)^a^	23 (13.1)^a, b^	3 (2.8)^a, b^	< 0.001
Systolic blood pressure		0 (0.0)						
Cardiac arrest	12 (2.5)		9 (15.8)^a, b, c^	0 (0.0)	1 (1.0)^a^	1 (0.6)^b^	1 (0.9)^c^	< 0.001
Unmeasurable	29 (6.0)		8 (14.0)	2 (5.6)	4 (3.8)	12 (6.9)	3 (2.8)	0.063
40–90 mmHg	98 (20.4)		5 (8.8)^a^	8 (22.2)	21 (20.2)	33 (18.9)	31 (28.4)^a^	0.045
> 90 mmHg	342 (71.1)		35 (61.4)	26 (72.2)	78 (75.0)	129 (73.7)	74 (67.9)	0.345
Heart rate, Median (IQR)	65 (49–84)	2 (0.4)	60 (35–99)	74 (49–95)	71 (58–87)^a^	63 (50–81)	60 (45–75)^a^	0.021
Glasgow coma scale, Median (IQR)	11 (8–14)	58 (12.1)	11 (6–13)	11 (8–13)	11 (8–14)	12 (9–14)	10 (7–14)	0.073
Biological data								
Serum pH, Median (IQR)	7.312 (7.246–7.368)	62 (12.9)	7.267 (7.178–7.318)^a, b^	7.312 (7.257–7.338)	7.306 (7.209–7.358)	7.329 (7.255–7.379)^a^	7.336 (7.273–7.375)^b^	0.001
Serum HCO_3_- (mEq/L), Median (IQR)	21 .0 (15.8–25.7)	66 (13.7)	19.9 (14.6–23.9)^a^	18.3 (15.1–25.6)	19.2 (14.4–24.0)^b^	21.9 (16.0–26.2)	22.8 (17.0–26.4)^a, b^	0.014
Serum Lactate (mmol/L), Median (IQR)	2.8 (1.3–6.0)	100 (20.8)	4.4 (2.2–8.8)^a, b^	3.1 (1.9–5.8)^c^	4.1 (2.0–7.0)^d, e^	2.1 (1.2–5.6)^a, d^	1.8 (0.9–3.6)^b, c, e^	< 0.001
Serum Sodium (mEq/L), Median (IQR)	140 (135–143)	7 (1.5)	140 (137–143)	141 (139–143)	140 (137–143)	139 (136–143)	138 (133–143)	0.272
Serum Potassium (mEq/L), Median (IQR)	4 (3.6–4.7)	6 (1.2)	4.2 (3.6–4.9)	4.2 (3.6–4.5)	4.1 (3.6–4.8)	4.0 (3.5–4.6)	4.0 (3.6–4.5)	0.891
Serum Glucose (mg/dL), Median (IQR)	127 (91–189)	44 (9.1)	164 (107–256)	122 (107–165)	132 (97–201)	127 (96–182)	109 (82–180)	0.051
SOFA score**	5 (3–7)	32 (13.0)	7 (3–9)	5 (4–7)	4 (2–6)	5 (3–7)	6 (3–8)	0.108
Cold exposure	378 (78.6)	13 (2.7)	51 (89.5)^a^	34 (94.4)^b^	90 (86.5)^c^	134 (76.6)	69 (63.3)^a, b, c^	< 0.001
Associated condition								
Internal disease	248 (51.6)	0 (0.0)	23 (40.4)^a^	13 (36.1)^b^	51 (49.0)	89 (50.9)	72 (66.1)^a, b^	0.003
Trauma	64 (13.3)	0 (0.0)	5 (8.8)	8 (22.2)	15 (14.4)	23 (13.1)	13 (11.9)	0.452
Alcohol intoxication	43 (8.9)	0 (0.0)	5 (8.8)	2 (5.6)	12 (11.5)	20 (11.4)	4 (3.7)	0.146
Drowning	27 (5.6)	0 (0.0)	8 (14.0)^a^	3 (8.3)	8 (7.7)	7 (4.0)	1 (0.9)^b^	0.004
Self-harm	30 (6.2)	0 (0.0)	6 (10.5)	5 (13.9)	9 (8.7)	8 (4.6)	2 (1.8)	0.017
other	126 (26.2)	0 (0.0)	14 (24.6)	7 (19.4)	24 (23.1)	48 (27.4)	33 (30.3)	0.665
Admission ward								
No admission	15 (3.1)	0 (0.0)	0 (0.0)	1 (2.8)	4 (3.8)	6 (3.4)	4 (3.7)	0.692
General ward	219 (45.5)	0 (0.0)	25 (43.9)	9 (25.0)	45 (43.3)	82 (46.9)	58 (53.2)	0.056
Intensive care unit	247 (51.4)	0 (0.0)	32 (56.1)	26 (72.2)^a^	55 (52.9)	87 (49.7)	47 (43.1)^a^	0.039
Rewarming procedure								
Active external or Minimally invasive	460 (95.6)	0 (0.0)	55 (96.5)	34 (94.4)	98 (94.2)	168 (96.0)	105 (96.3)	0.917
Warm intravenous fluids	360 (74.8)	0 (0.0)	51 (89.5)^a^	30 (83.3)	83 (79.8)^b^	130 (74.3)	66 (60.6)^a, b^	< 0.001
Warm blanket	340 (70.7)	0 (0.0)	28 (49.1)^a, b^	20 (55.6)^c^	67 (64.0)	137 (78.3)^a^	88 (80.7)^b, c^	< 0.001
Forced warm air	76 (15.8)	0 (0.0)	13 (22.8)^a^	9 (25.0)	22 (21.2)	24 (13.7)	8 (7.3)^a^	0.007
Heating Pads	20 (4.2)	0 (0.0)	4 (7.0)	4 (11.1)	7 (6.7)	2 (1.1)	3 (2.8)	0.009
Warm bath	15 (3.1)	0 (0.0)	3 (5.3)	5 (13.9)^a, b^	5 (4.8)	1 (0.6)^a^	1 (0.9)^b^	< 0.001
Active internal	80 (16.6)	0 (0.0)	19 (33.3)^a^	6 (16.7)	16 (15.4)	28 (16.0)	11 (10.1)^a^	0.009
Lavage	38 (7.9)	0 (0.0)	5 (8.8)	3 (8.3)	11 (10.6)	13 (7.4)	6 (5.5)	0.708
Intravascular	4 (0.8)	0 (0.0)	0 (0.0)	1 (2.8)	0 (0.0)	2 (1.1)	1 (0.9)	0.496
Hemodialysis	25 (5.2)	0 (0.0)	2 (3.5)	1 (2.8)	5 (4.8)	13 (7.4)	4 (3.7)	0.675
ECMO	18 (3.7)	0 (0.0)	13 (22.8)^a, b, c^	2 (5.6)	1 (1.0)^a^	1 (0.6)^b^	1 (0.9)^c^	< 0.001
Other treatment								
Intubation	29 (6.03)	0 (0.0)	6 (10.5)	2 (5.6)	6 (5.8)	10 (5.7)	5 (4.6)	0.663
Catecholamine	82 (17.1)	0 (0.0)	16 (28.1)	12 (33.3)^a^	12 (11.5)^a^	23 (13.1)	19 (17.4)	0.005
Emergent transvenous cardiac pacing	6 (1.3)	0 (0.0)	1 (1.8)	2 (5.6)	0 (0.0)	1 (0.6)	2 (1.8)	0.075
Rewarming rate (°C/h), Median (IQR)^†^	0.85 (0.53–1.31)	0 (0.0)	2.7 (2.25–3.8)	1.68 (1.6–1.84)	1.20 (1.09–1.31)	0.73 (0.59–0.85)	0.34 (0.25–0.41)	< 0.001

Values are expressed as numbers (percentages) unless indicated otherwise

Groups that share a superscript letter were significantly different from post-hoc pairwise comparisons

*SOFA* sequential organ failure assessment, *ECMO* extracorporeal membrane oxygenation

* Represents P for heterogeneity across the 5 rewarming rate groups. Comparisons between the 5 groups were evaluated with Kruskal-Wallis test for numeric variables and Fisher’s exact test for categorical variables. For post-hoc pairwise comparisons of these tests, Steel-Dwass multiple comparison tests and Bonferroni correction for multiple comparisons were used, respectively

** Calculated with patients admitting to intensive care units

^†^ Post-hoc analysis was omitted

### Outcome

The outcomes of this study are presented in Table 3. The in-hospital mortality rates were 19.3% (11/57), 11.1% (4/36), 14.4% (15/104), 20.1% (35/175), and 34.9% (38/109) in the ≥2.0 °C/h, 1.5–< 2.0 °C/h, 1.0–< 1.5 °C/h, 0.5–< 1.0 °C/h, and < 0.5 °C/h groups, respectively. Multivariate regression analysis revealed that the in-hospital mortality rate increased with each 0.5 °C/h decrease in RR (AOR, 1.49; 95% CI, 1.15–1.94; *P*_trend_ < 0.01). Additionally, the mortality rate was significantly higher in the < 0.5 °C/h group than in the ≥2.0 °C/h group (AOR, 4.09; 95% CI, 1.33–12.6). Regarding the case of the secondary outcome, univariate logistic analysis revealed that the incidence of VF/VT decreased with each 0.5 °C/h decrease in RR (AOR, 0.55; 95% CI, 0.33–0.90; *P*_trend_ = 0.016). According to the subgroup analysis, although each analysis showed heterogeneity and under power, the negative association of RR and mortality was constant (Table 4).

**Table 3:** In-hospital mortality and the incidence of VF or pulseless VT

	All patients	Rewarming rate (°C/h)					Odds for trend	P for trend
		(≥2.0)	(1.5–< 2.0)	(1.0–< 1.5)	(0.5–< 1.0)	(< 0.5)		
	*n* = 481	*n* = 57	*n* = 36	*n* = 104	*n* = 175	*n* = 109		
Primary outcome								
In-hospital mortality	103 (21.4)	11 (19.3)	4 (11.1)	15 (14.4)	35 (20)	38 (34.9)		
Odds ratio (95% CI)		Reference	0.52 (0.15–1.79)	0.71 (0.30–1.66)	1.05 (0.50–2.24)	2.24 (1.04–4.82)	1.33 (1.10–1.61)	0.003
Adjusted odds ratio (95% CI)*		Reference	1.11 (0.27–4.62)	1.23 (0.41–3.66)	1.83 (0.64–5.22)	4.09 (1.33–12.6)	1.49 (1.15–1.94)	0.003
Secondary Outcome								
Vf or pulseless VT during rewarming procedure	9 (1.9)	2 (3.5)	2 (5.6)	4 (3.8)	1 (0.6)	0 (0.0)		
Odds ratio (95% CI)		Reference	1.62 (0.22–12.0)	1.10 (0.20–6.20)	0.16 (0.01–1.78)	N/A	0.55 (0.33–0.90)	0.016

Values are expressed as numbers (percentages) unless indicated otherwise

*Vf* ventricular fibrillation, *VT* ventricular tachycardia, *CI* confidence interval

* Adjusted for sex, age category, body temperature at arrival to the hospital, the number of past medical history, activity of daily living, cold exposure, systolic blood pressure, internal disease etiology, active internal rewarming procedure

**Table 4:** In-hospital mortality (subgroup analysis)

	All patients	Rewarming rate (°C/h)					Odds for trend	P for trend
		(≥2.0)	(1.5–2.0)	(1.0–1.5)	(0.5–1.0)	(< 0.5)		
	*n* = 481	*n* = 57	*N* = 36	*n* = 104	*n* = 175	*n* = 109		
Age category								
Adults aged 18–64 years	101	15	8	27	35	16		
In-hospital mortality	12 (11.9)	1 (6.7)	1 (12.5)	3 (11.1)	5 (14.3)	2 (12.5)		
Odds ratio (95% CI)		Reference	2.00 (0.11–37.0)	1.75 (0.17–18.5)	2.33 (0.25–21.9)	2.00 (0.16–24.7)	1.17 (0.71–1.95)	0.532
Adjusted odds ratio (95% CI)^a^		Reference	N/A	N/A	N/A	N/A	1.69 (0.87–3.30)	0.124
Elderly patients aged ≥65 years	380	42	28	77	140	93		
In-hospital mortality (%)	91 (23.9)	10 (23.8)	3 (10.7)	12 (15.6)	30 (21.4)	36 (38.7)		
Odds ratio (95% CI)		Reference	0.38 (0.10–1.55)	0.59 (0.23–1.51)	0.87 (0.39–1.98)	2.02 (0.89–4.61)	1.33 (1.08–1.64)	0.008
Adjusted odds ratio (95% CI)^a^		Reference	0.68 (0.15–3.17)	0.83 (0.27–2.55)	1.49 (0.51–4.37)	3.68 (1.15–11.8)	1.53 (1.15–2.04)	0.004
Location								
Indoor	375	36	27	68	150	94		
In-hospital mortality (%)	94 (25.1)	10 (27.8)	3 (11.1)	13 (19.1)	32 (21.3)	36 (38.3)		
Odds ratio (95% CI)		Reference	0.33 (0.08–1.32)	0.62 (0.24–1.58)	0.71 (0.31–1.61)	1.61 (0.70–3.74)	1.25 (1.01–1.54)	0.039
Adjusted odds ratio (95% CI)^b^		Reference	0.55 (0.11–2.68)	0.89 (0.27–2.87)	1.03 (0.34–3.10)	2.48 (0.76–8.14)	1.36 (1.03–1.80)	0.033
Outdoor	106	21	9	36	25	15		
In-hospital mortality (%)	9 (8.5)	1 (4.8)	1 (11.1)	2 (5.6)	3 (12.0)	2 (13.3)		
Odds ratio (95% CI)		Reference	2.50 (0.14–45.0)	1.18 (0.10–13.8)	2.73 (0.26–28.4)	3.08 (0.25–37.5)	1.32 (0.75–2.32)	0.330
Adjusted odds ratio (95% CI)^b^		Reference	N/A	N/A	N/A	N/A	1.98 (0.58–6.76)	0.273
Body temperature at arrival to the hospital								
Mild (≥32 °C)	163	10	5	27	60	61		
In-hospital mortality (%)	32 (19.6)	0 (0.0)	1 (20.0)	3 (11.1)	6 (10.0)	22 (36.1)		
Odds ratio (95% CI)		Reference	N/A	N/A	N/A	N/A	2.43 (1.38–4.25)	0.002
Adjusted odds ratio (95% CI)^c^		Reference	N/A	N/A	N/A	N/A	2.21 (1.15–4.25)	0.017
Moderate (28–32 °C)	216	15	16	48	92	45		
In-hospital mortality (%)	45 (20.8)	2 (13.3)	1 (6.2)	6 (12.5)	21 (22.8)	15 (33.3)		
Odds ratio (95% CI)		Reference	0.43 (0.04–5.35)	0.93 (0.17–5.17)	1.92 (0.40–9.21)	3.25 (0.65–16.3)	1.62 (1.13–2.32)	0.009
Adjusted odds ratio (95% CI)^c^		Reference	0.79 (0.05–11.9)	0.82 (0.12–5.62)	1.69 (0.28–10.2)	2.86 (0.43–18.9)	1.48 (0.96–2.28)	0.078
Severe (< 28 °C)	102	32	15	29	23	3		
In-hospital mortality (%)	26 (25.5)	9 (28.1)	2 (13.3)	6 (20.7)	8 (34.8)	1 (33.3)		
Odds ratio (95% CI)		Reference	0.39 (0.07–2.10)	0.67 (0.20–2.18)	1.36 (0.43–4.32)	1.28 (0.10–15.9)	1.10 (0.76–1.58)	0.612
Adjusted odds ratio (95% CI)^c^		Reference	0.49 (0.07–3.44)	0.58 (0.12–2.90)	1.18 (0.23–6.08)	2.44 (0.13–47.5)	1.17 (0.71–1.93)	0.548
Associated condition								
Patients with internal disease etiology	248	23	13	51	89	72		
In-hospital mortality (%)	71 (28.6)	5 (21.7)	3 (23.1)	12 (23.5)	22 (24.7)	29 (40.3)		
Odds ratio (95% CI)		Reference	1.08 (0.21–5.49)	1.11 (0.34–3.62)	1.18 (0.39–3.56)	2.43 (0.81–7.27)	1.29 (1.00–1.65)	0.047
Adjusted odds ratio (95% CI)^d^		Reference	2.04 (0.30–13.8)	1.50 (0.36–6.24)	1.57 (0.40–6.22)	3.48 (0.81–15.0)	1.33 (0.96–1.83)	0.085
Patients without internal disease etiology	233	34	23	53	86	37		
In-hospital mortality (%)	32 (13.7)	6 (17.6)	1 (4.3)	3 (5.7)	13 (15.1)	9 (24.3)		
Odds ratio (95% CI)		Reference	0.21 (0.02–1.89)	0.28 (0.07–1.21)	0.83 (0.29–2.40)	1.50 (0.47–4.78)	1.23 (0.90–1.68)	0.202
Adjusted odds ratio (95% CI)^d^		Reference	0.59 (0.04–8.37)	1.17 (0.14–9.54)	3.96 (0.56–28.2)	10.0 (1.13–88.8)	2.07 (1.23–3.48)	0.006
Rewarming Procedure								
Patients with active internal rewarming	80	19	6	16	28	11		
In-hospital mortality (%)	21 (26.3)	8 (42.1)	1 (16.7)	2 (12.5)	5 (17.9)	5 (45.5)		
Odds ratio (95% CI)		Reference	0.28 (0.03–2.83)	0.20 (0.03–1.12)	0.30 (0.08–1.13)	1.15 (0.26–5.11)	0.89 (0.62–1.27)	0.513
Adjusted odds ratio (95% CI)^e^		Reference	0.26 (0.01–5.37)	0.23 (0.01–3.74)	0.33 (0.03–4.44)	3.42 (0.26–45.3)	1.29 (0.71–2.36)	0.408
Patients without active internal rewarming	401	38	30	88	147	98		
In-hospital mortality (%)	82 (20.4)	3 (7.9)	3 (10.0)	13 (14.8)	30 (20.4)	33 (33.7)		
Odds ratio (95% CI)		Reference	1.30 (0.24–6.94)	2.02 (0.54–7.55)	2.99 (0.86–10.4)	5.92 (1.69–20.7)	1.63 (1.27–2.08)	< 0.001
Adjusted odds ratio (95% CI)^e^		Reference	1.52 (0.24–9.49)	1.86 (0.43–8.08)	2.91 (0.70–12.1)	6.14 (1.38–27.4)	1.63 (1.19–2.25)	0.002

Values are expressed as numbers (percentages) unless indicated otherwise

^a^Adjusted for sex, body temperature at arrival to the hospital, the number of past medical history, activity of daily living, cold exposure, systolic blood pressure, internal disease etiology, active internal rewarming procedure

^b^Adjusted for sex, age category, body temperature at arrival to the hospital, the number of past medical history, activity of daily living, cold exposure, systolic blood pressure, internal disease etiology, active internal rewarming procedure

^c^Adjusted for sex, age category, the number of past medical history, activity of daily living, cold exposure, systolic blood pressure, internal disease etiology, active internal rewarming procedure

^d^Adjusted for sex, age category, body temperature at arrival to the hospital, the number of past medical history, activity of daily living, cold exposure, systolic blood pressure, active internal rewarming procedure

^e^Adjusted for sex, age category, body temperature at arrival to the hospital, the number of past medical history, activity of daily living, cold exposure, systolic blood pressure, internal disease etiology

## Discussion

In this study, we found that the RR of patients with AH is independently associated with mortality after adjusting the important potentially confounding factors. In-hospital mortality rates increase with each 0.5 °C/h decrease in RR. Furthermore, the mortality rate in the < 0.5 °C/h group was significantly higher than that in the > 2.0 °C/h group. To the best of our knowledge, this is the first multicentre study to assess the association between RR and mortality, and the findings of this study may provide important information regarding the appropriate treatment of AH.

We initially show that patients with slower RR have the following characteristics: are more likely to be older, have lower body temperature at arrival to the hospital, and have an internal disease. This result is consistent with that of the previous studies [6, 17]. However, the RR in this study was slower than that in the previous studies because of the older age of our study population [10]. Another possible reason is the high proportion of the patients who developed AH in an indoor setting, which might be the result of underlying diseases such as infections [18].

Previous studies have suggested a possible association between RR and mortality. Daniel et al. reported that the mean RR in patients with AH who died was significantly slower than that in surviving patients in a retrospective multicentre observational study [17]. Kathleen et al. reported that the likelihood of mortality was associated with slower RR in a single-centre observational study, which included 96 patients [18]. These results are, although not the main outcome of the studies and derived from univariate analysis, consistent with the results of our study. In this study, we confirmed the negative association between RR and mortality using multivariate logistic analysis with 481 patients, which is the largest sample size in this field.

There are several known physiological effects of hypothermia that could result in high mortality. Cardiac contractility and pulse rate decrease as the heart cools [1, 3, 8]. Cold stress reduces the circulating blood volume due to cold-induced diuresis, extravascular plasma shift, and inadequate fluid intake [6, 19, 20]. Both hypo- and hyper-kalaemia occur in patients with AH due to the shift of extracellular potassium into the cells, acidosis, and cell death [8, 21]. Ventilatory response to carbon dioxide is attenuated and results in respiratory acidosis [3, 22]. These cardiorespiratory effects may lead to the clinical manifestations of shock and dysrhythmia [19, 22]. Cough reflex is obtunded, and ciliary activity is reduced [1, 8], predisposing to aspiration and pneumonia. Coagulopathy also occurs and is critical for patients with AH with severe trauma [23, 24]. For patients with AH, slower RR means prolonged periods of susceptibility to these potentially harmful physiological effects and could result in a higher incidence of mortality.

Another outcome of this study is the incidence of VF/VT. Because VF/VT in patients with AH may be unresponsive to anti-arrhythmic drug and defibrillation [1, 7, 19, 25], they are the most critical complication during rewarming. Our data showed a positive association between RR and VF/VT. However, the incidence of VF/VT was highest in the RR of the 1.5–< 2.0 °C/h group and not in the fastest RR group. Both rapid rewarming accompanied by rapid or unpredictable changes in myocardial temperature and slow rewarming accompanied by prolonged low core body temperature can be the predisposing factors of VT/VF [26, 27]. These factors may be counterbalanced, and thus, the occurrence of VT/VF in our study increased in the high-intermediate RR group. As the number of patients who presented with VF/VT was significantly small to draw a conclusion, further study is required to confirm the association between RR and the incidence of VF/VT to establish more appropriate rewarming strategies.

The selection of the rewarming method can vary since no rewarming method has been proven to be better than the other rewarming methods. Our study has shown that regardless of the rewarming method, the RR is associated with mortality. It is reasonable to choose active internal rewarming when rewarming patients is urgent because of the haemodynamic instability due to severe hypothermia [5, 28–31]. However, considering the potential risk of complications with invasive rewarming methods [1], as well as the insufficient evidence that these methods improve the outcome in all patients with severe hypothermia, the best approach, not only for patient outcomes but also for healthcare cost, may be the stepwise approach that begins with active external and minimally invasive rewarming, and saving invasive method for patients who cannot be rewarmed adequately.

This study has several limitations. First, this was a retrospective chart review study; hence, missing data were unavoidable. A total of 54 patients were excluded because of missing data, but the number of patients included in this study is still the largest in this field. Second, we could not categorise patients whose RR was over 2.0 °C/h because of the small number of these patients. Thus, we plan to conduct a prospective study to obtain a larger number of patients. Third, although we have adjusted for the possible confounding factors, there may be residual confounding factors because of the retrospective design. Fourth, the proportion of ‘cold exposure’ was approximately 80% in this study. However, the possibility of underestimation of cold exposure could not be ruled out because we retrospectively obtained the data of this registry.

## Conclusion

In this study, we found that overall, in-hospital mortality rates increase with each 0.5 °C/h decrease in RR. However, judging from the results of subgroup analyses, the safest RR might differ according to the patient status or rewarming methods.

## Data Availability

The datasets used and/or analysed during the current study are available from the corresponding author on reasonable request.
